# Measurement of extracapsular extension in sentinel lymph node as a possible predictor of residual axillary disease in breast cancer

**DOI:** 10.1016/j.clinsp.2023.100216

**Published:** 2023-05-16

**Authors:** Gabriela Boufelli de Freitas, Bruna Salani Mota, Jonathan Yugo Maesaka, Cintia Cardoso Pinheiro, Luiz Guilherme Cernaglia Aureliano de Lima, José Maria Soares, Edmund Chada Baracat, José Roberto Filassi

**Affiliations:** Setor de Mastologia da Disciplina de Ginecologia do Departamento de Obstetricia e Ginecologia, Hospital das Clínicas, Faculdade de Medicina da Universidade de São Paulo (FMUSP), Av. Dr. Arnaldo, 251; 4o andar Secretária Cirúrgica, São Paulo, SP 01246-000, Brazil

**Keywords:** Breast Neoplasms, Biopsy, Sentinel Lymph Node, Lymphatic metastasis

## Abstract

**Background:**

The presence of Extracapsular Extension (ECE) in the Sentinel Lymph Node Biopsy (SLNB) is still a doubt in the literature. Some studies suggest that the presence of ECE may be related to a greater number of positive axillary lymph nodes which could impact Disease Free Survival (DFS) and Overall Survival (OS). This study searches for the clinical significance of the ECE.

**Methods:**

Retrospective cohort comparing the presence or absence of ECE in T1-2 invasive breast cancer with positive SLNB. All cases treated surgically at the Cancer Institute of the State of São Paulo (ICESP) between 2009 and 2013 were analyzed. All patients with axillary disease in SLNB underwent AD.

**Outcomes:**

Identify the association between the presence and length of ECE and additional axillary positive lymph nodes, OS and DFS between both groups.

**Results:**

128 patients with positive SLNB were included, and 65 had ECE. The mean metastasis size of 0.62 (SD = 0.59) mm at SLNB was related to the presence of ECE (p < 0.008). The presence of ECE was related to a higher mean of positive sentinel lymph nodes, 3.9 (± 4.8) vs. 2.0 (± 2.1), p = 0.001. The median length of follow-up was 115 months. The OS and DFS rates had no differences between the groups.

**Conclusion:**

The presence of ECE was associated with additional positive axillary lymph nodes in this study. Therefore, the OS and DFS were similar in both groups after 10 years of follow-up. It is necessary for additional studies to define the importance of AD when SLNB with ECE.

## Highlights

• This study aimed to find the clinical significance of the extracapsular extension in sentinel lymph nodes on the number of additional involved axillary lymph nodes, overall survival, and disease-free survival.
• One hundred twenty-eight patients with positive sentinel lymph nodes were separated into two groups according to the presence or absence of ECE and following during 10 years.
• There was found a positive correlation between the presence of ECE and the number of additional positive lymph nodes. Therefore, this does not impact overall survival, and disease-free survival in a follow-up of 10 years.

## Introduction

Surgical breast cancer treatment has undergone significant changes in the last decade. The surgical field adopting de-escalation measures allows patients to control the disease with less morbidity from more aggressive treatments.^1^, ^2^, ^3^, ^4^, ^5^, ^6^

The Axillary Dissection (AD) was performed on all patients until the ACOSOG Z0011 study revealed that patients with early breast cancer (T1/T2) undergoing breast-conserving surgery with up to 2 compromised Sentinel Lymph Node Biopsy (SLNB) could avoid AD with no impact on overall survival after a follow-up of 10 years.[7] This practice-change clinical trial did not include participants with Extracapsular Extension (ECE) in the SLNB.

ECE is defined by tumor invasion of the lymph node capsule or the tumor passing through the nodal capsule into the perinodal tissue. Its extracapsular involvement is considered in the literature as a risk factor for the presence of disease in other axillary lymph nodes.[2,8,9]

Only one study evaluated ECE-positive SLNB and showed that there is a 20% greater chance of other positive lymph nodes when the extracapsular invasion is present, therefore there is no mention of the impact on survival outcomes in this cohort.[10] However, there is still a gap in the literature to guide better decision-making of axillary surgical approaches. The clinical decision to perform or not axillary dissection is frequently based on opinions related to the chance of having an additional axillary disease.[11]

This study aimed to assess if the presence of ECE in positive SLNB was associated with axillary tumor burden in early breast cancer and its impact on overall survival and disease-free survival.

## Methods

This study was a retrospective cohort study with convenience sampling following the STROBE guideline comparing the presence or absence of Extracapsular Extension (ECE) in invasive breast cancer with positive sentinel lymph node biopsy.

The inclusion criteria were T1 and T2 invasive breast cancer with positive SLNB by routine Hematoxylin and Eosin (H&E) staining treated surgically at the Cancer Institute of the State of São Paulo (ICESP) between February 2009 and December 2013 were analyzed. The exclusion criteria were neoadjuvant chemotherapy treatment or those with pathologically negative SLNB and T3 tumors. The Local Ethics Committee evaluated and approved this research (number 395/15).

All patients underwent intraoperative frozen SLNB, a routine in the Institution; when it was positive, the AD was performed in the same surgery. When the involvement of the SLNB was detected in the paraffin exam, the patient underwent a new surgery for AD. The SLNB and AD technique was previously described in other publications.[12]

The presence and extent of ECE in the SLNB, are defined as absence or presence. When H&E detected the presence of ECE, it was measured in millimeters. All pathological findings and missing data were reviewed by a breast-specialized pathologist.

### Statistical analysis

The variables were analyzed using measures of continuous central tendency (including mean and median) and measures of dispersion. The Kolmogorov-Smirnov and Shapiro-Wilk tests were applied to assess data distribution characteristics. To compare outcomes with categorical variables was used the Chi-Square test or Fisher's exact test. Continuous data were analyzed by the nonparametric Mann-Whitney *U*-test. The Spearman's association was used to assess the correlation between the size of the metastasis and the presence or absence of extracapsular invasion with the presence of other compromised axillary lymph nodes. The time-to-event outcomes (local recurrence and overall survival) were analyzed using the Kaplan-Meyer survival function and the Log-Rank test. The follow-up losses and deaths were censored. The data were analyzed using the SPSS v20.0 program. For all tests, a significance level of 5% was considered.

## Results

A total of 128 patients with positive sentinel lymph node biopsy by H&E staining were included, of which 65 had an extracapsular extension in the sentinel lymph node. Of 137 patients, nine were excluded due to they were locally advanced tumors.

**Fig. 1:**
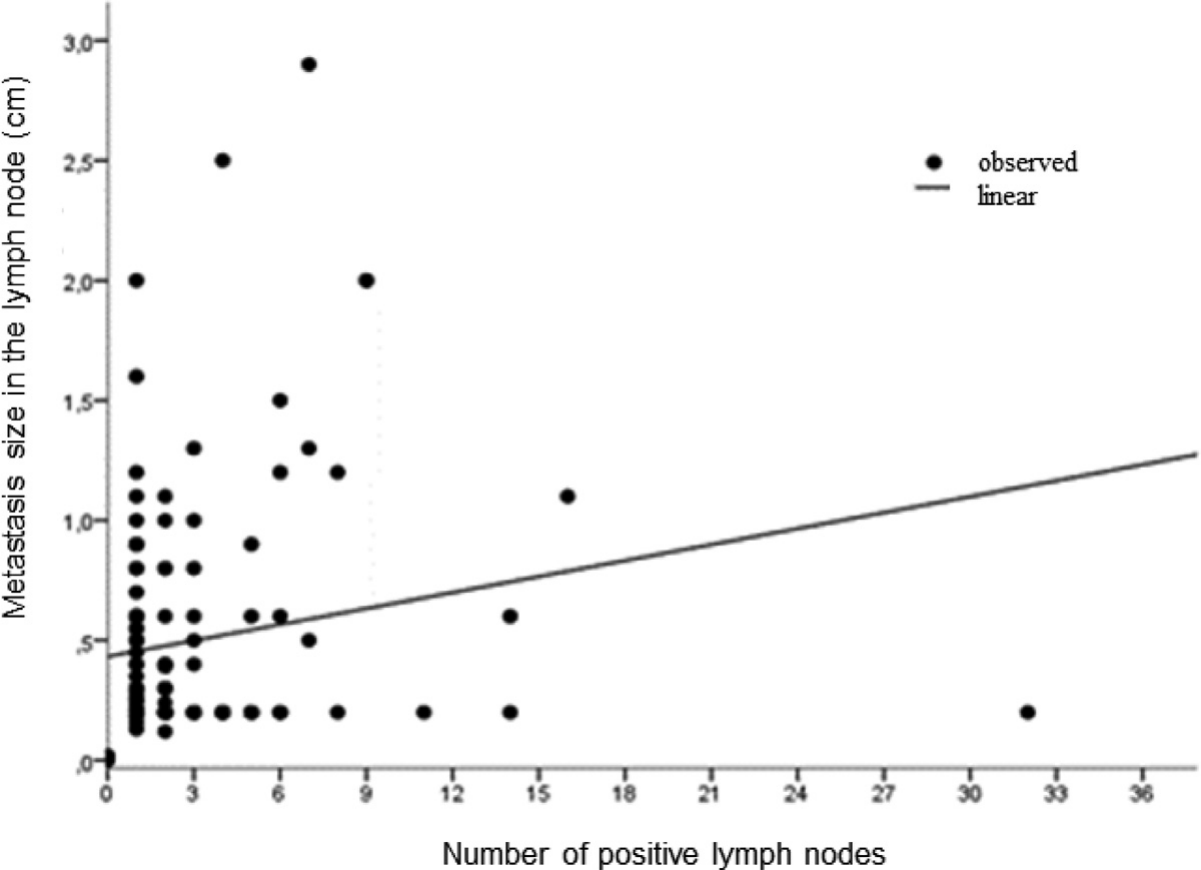
Number of positive lymph nodes according to the measurement of metastatic lymph node involvement.

The clinical and anatomopathological characteristics of the included patients are described in Table 1. 39.8% (51/128) of patients had tumors up to 2 cm and 60.2% (77/128) had tumors between 2 to 5 cm. 75% (96/128) had up to 3 affected lymph nodes. The size of the breast Tumor (pT) was related to ECE, 35.2% vs. 25% were pT2 in the group with ECE vs. without ECE, respectively (p = 0.033). The number of pN2 was also statistically different (14.1% vs. 5.5%, p = 0.0001) between the group with and without ECE. The size of the metastasis measured by microscopy was related to the lymph node involvement (Fig. 1). The median size was 0.62 cm in the presence of ECE versus 0.37 cm in the absence of ECE (p = 0.008) (Table 2). There was no difference in anatomopathological characteristics, histological subtype, molecular subtype, nuclear grade, histological grade, or lymphovascular invasion between the groups with and without extracapsular involvement, as described in Table 1.

**Table 1:** Clinical and pathological characteristics of patients.

			**ECE**		**Total**	**p**
			**Absence**	**Presence**		
Age (mean)			57,9	57,4		0.33
Lymphovascular invasion	Absence	n (%)	43 (33.86)	37 (29.13)	81 (62.99)	0.15
	Presence	n (%)	19 (14.96)	28 (22.05)	47 (37.01)	
Nuclear grade	1	n (%)	1 (0.78)	4 (3.12)	5 (3.91)	0.334
	2	n (%)	33 (25.78)	29 (22.66)	62(48.44)	
	3	n (%)	29 (22.66)	32 (25.00)	61 (47.66)	
Histologic grade	1	n (%)	13 (10.16)	13 (10.16)	26 (20.31)	0.89
	2	n (%)	34 (25.56)	30 (23.44)	64 (50.00)	
	3	n (%)	16 (12.50)	22 (17.19)	38 (29.69)	
Pt ‒ TNM	T1	n (%)	31 (24.22)	20 (15.62)	51 (39.84)	0.03
	T2	n (%)	32 (25.00)	45 (35.16)	77 (60.16)	
Pn ‒ TNM	N0 (i+)	n (%)	2(1.56)	0 (0.0)	2(1.56)	0.02
	N1	n (%)	53 (41.41)	43 (33.59)	96 (75.0)	
	N2	n (%)	7 (5.47)	18 (14.06)	25 (19.53)	
	N3	n (%)	1 (0.78)	4 (3.12)	5 (3.91)	
Histologic type	Idc	n (%)	58 (45.31)	60 (48.88)	118 (92.19)	0.79
	Ilc	n (%)	3 (2.34)	4 (3.12)	7 (5.47)	
	Other	n (%)	2 (1.56)	1 (0.78)	3 (2.34)	
Molecular subtype	Luminal A	n (%)	30 (23.44)	29 (22.66)	59 (46.09)	0.76
	Luminal B	n (%)	20 (15.62)	26 (20.31)	46 (35.94)	
	Rh-/her2-	n (%)	5 (3.91)	4 (3.12)	9 (7.03)	
	Rh+/her2+	n (%)	5 (3.91)	5 (3.91)	10 (7.81)	
	Rh-/her2+	n (%)	3 (2.34)	1 (0.78)	4 (3.12)	

Pearson's Qui-Square.

IDC, Invasive Ductal Carcinoma; ILC, Invasive Lobular Carcinoma; i+, Isolated Tumor Cell.

**Table 2:** Metastasis size assessment of SLNB and positive LND according to the presence of ECE.

	**ECE**	**n**	**Mean (Standard Deviation)**	**p**
Metastasis size in the SLN (cm)	Absence	63	0.3676 (0.32)	0.008
	Presence	65	0.6248 (0.59)	
Number of positive LND	Absence	63	2.000 (2.063)	0.001
	Presence	65	3.877 (4.784)	
*t*-test				

SLN, Sentinel Lymph Node; ECE, Extracapsular Extension.

**Fig. 2:**
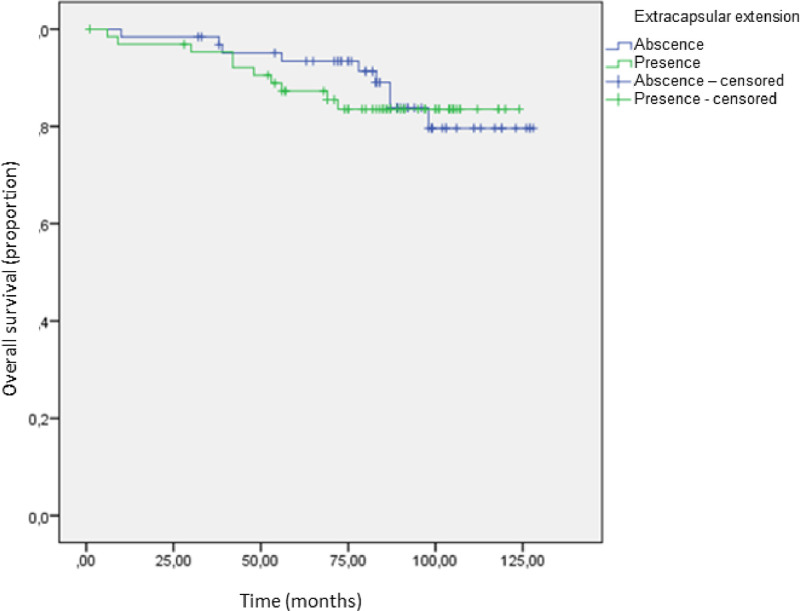
Overall survival according to the presence of extracapsular extension.

There was a correlation between the presence or absence of ECE and the mean number of lymph nodes involved 3.9 vs. 2.0, p = 0.00; respectively (Table 2). Therefore, evaluating the size of the extracapsular involvement, no difference was found between ≥ or < 2 mm in the number of lymph nodes involved (p = 0.44) and there is no linear correlation (Fig. 2).

The median length follow-up was 114 months (range 108‒122 months) versus 113 months (range 106‒120 months) in absent ECE and present ECE, respectively. There was no difference in overall survival (Fig. 2). The overall survival rates were 84.6% (55/65 patients) in the group with ECE versus 85.7% (54/63 patients) without ECE (p = 0.761) (Table 3).

**Table 3:** Overall survival in both groups.

**ECE**	**n**	**Events (deaths)**	**Overall survival**	
			**n**	**%**
Absence	63	9	54	85.7%
Presence	65	10	55	84.6%
Total	128	19	109	85.2%
Follow-up 115 months				

ECE, Extracapsular Extension.

**Fig. 3:**
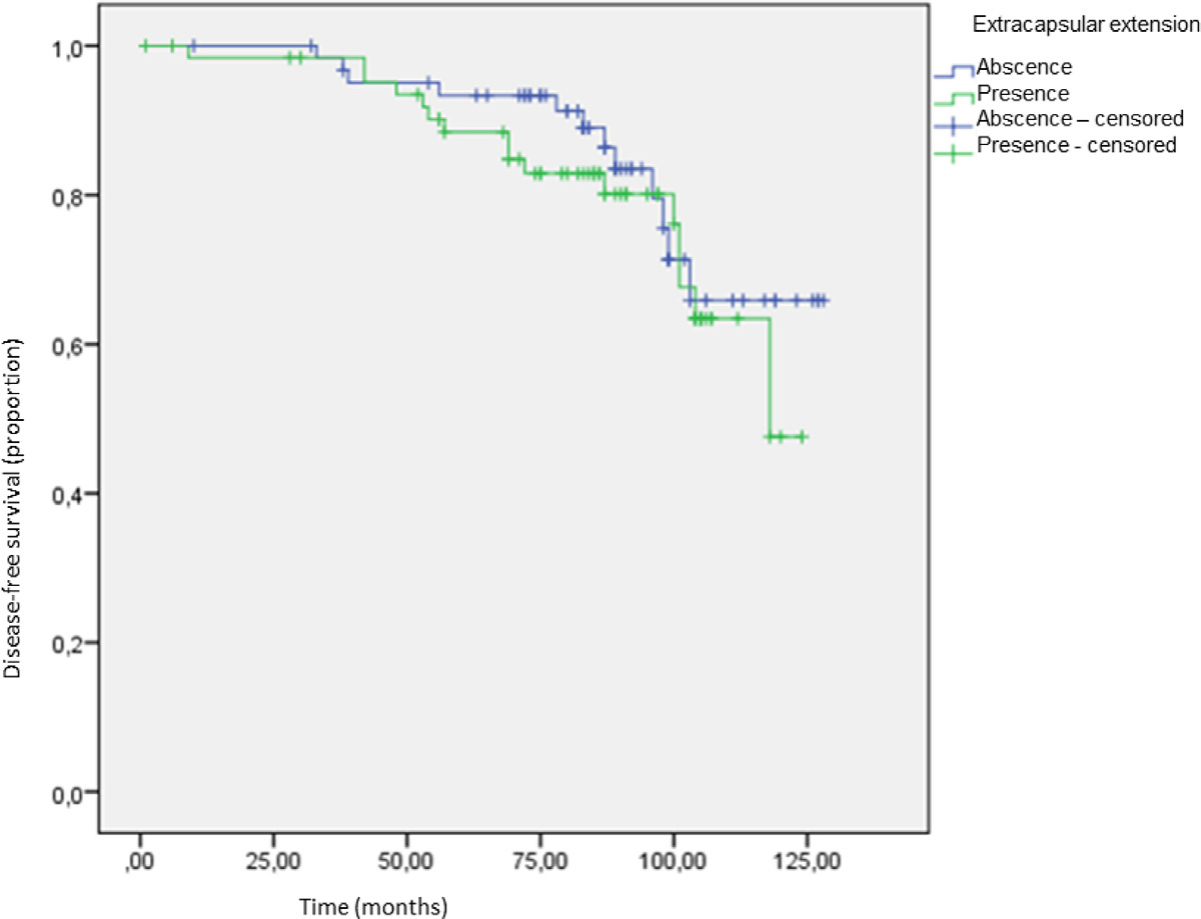
Disease-free survival according to the presence of extracapsular extension.

There was no difference in disease-free survival (Fig. 3). The disease-free survival rates were 81% (12 recurrences; 3 local recurrences and 9 visceral recurrences) in the absent ECE group versus 75.4% (16 recurrences; 1 local recurrence and 15 visceral recurrences) in present ECE group (p = 0.456) (Table 4).

**Table 4:** Disease-free survival in both groups.

**ECE**	**n**	**Local recurrence**	**Visceral recurrence**	**Disease-free survival**	
				**n**	**%**
Absence	63	3	9	51	81.0%
Presence	65	1	15	49	75.4%
Total	128	4	24	100	78.1%
Follow up 115 months					

ECE, Extracapsular Extension.

## Discussion

This retrospective analysis found a positive association between the presence of ECE and the number of lymph nodes involved in the axilla.

The association between lymph node metastasis size and axillary disease was also described in a meta-analysis published in 2011, which analyzed 56 studies that investigated predictive factors of lymph node involvement in addition to the sentinel lymph node.[13] In addition to the size of lymph node metastasis greater than 2 mm, the following factors were also identified as predictors of residual lymph node involvement: extracapsular extension at the sentinel lymph node, more than one positive SLN, tumor size >2 cm, lymphovascular invasion in the primary tumor and method of histological detection of metastasis in SLN. Ilknur et al. also found an association between metastasis size and axillary involvement when evaluating 221 patients with T1/T2 breast cancer.[14]

The present study found an association with the presence of ECE in tumors larger than 2 cm. In a study by Gooch et al., which evaluated 778 T1/T2 patients, N0 before surgery, with 1‒2 positive lymph nodes, 331 presented extracapsular extension. 180 with < 2 mm of involvement and 151 with > 2 mm. About 33% of patients with ECE > 2 mm had ≥ four positive lymph nodes in axillary dissection and only 9% when ECE < 2 mm; therefore, axillary dissection or axillary RT is recommended in cases with ECE > 2 mm.[10]

Recurrence-free survival and overall survival were similar in both groups in the present study with a 10-year follow-up. Ilknur et al., when evaluating 221 patients with T1/T2 breast cancer, found extracapsular involvement in 127 cases. The extracapsular extension had significant prognostic value for local and distant recurrence-free survival but had no impact on overall survival in 55 months follow-up.[14]

In this study, the fact that ECE did not impact overall survival in a 10-year follow-up suggests the possibility of applying the ACOSOG Z0011 criteria, without performing AD in a positive SLNB, even if it has ECE. Nevertheless, shared decision-making with improved clinicians' communication with patients is highly recommended, due to the lack of data and the possibility of further studies about positive sentinel lymph nodes with the presence of extracapsular extension could change these results.

According to Luciana Landeiro et al.[15] in Brazil, the rate of return to work following breast cancer therapy was 22%, 30%, and 60% after six, twelve, and twenty-four months, respectively, and this percentage was proportional to the aggressiveness of the treatment. The present research gives information that may lead to less extensive treatment and, as a consequence, fewer morbid procedures, resulting in a faster return to healthy activities. It is consistent with the main objective of oncological therapy, which is to increase breast cancer survivors' overall survival with less damaging consequences on their quality of life.

As a limitation of the study, the retrospective nature could overestimate the results; in addition, a larger sample of cases could increase the power of the study. Therefore, the study was carried out at a single center, a regional reference in oncology, which increases the homogeneity of conduct and has a considerably long follow-up. Finally, this study brings more information to a subject with limited data in the literature.

## Conclusion

The presence of ECE was associated with the number of affected axillary lymph nodes in this study. Therefore, overall survival and recurrence-free survival were similar in both groups, with a 10-year follow-up. It is necessary for additional studies to define the need for additional AD when SLNB identifies ECE.

## Authors’ contributions

Conceived and designed the analysis: GBF, JYM, CCP, LGCAL.

Collected the data: GBF, JYM, CCP, LGCAL.

Contributed data or analysis tools: GBF, BSM, JYM, CCP, LGCAL.

Performed the analysis: GBF, BSM, JYM, CCP, LGCAL, JMS, ECB, JRF.

Draft the paper: GBF, BSM, JYM, CCP, LGCAL, JMS, ECB, JRF.

Approved the final version: GBF, BSM, JYM, CCP, LGCAL, JMS, ECB, JRF.

## Declaration of Competing Interest

The authors declare no conflicts of interest.
